# Fine-grained parallel RNAalifold algorithm for RNA secondary structure prediction on FPGA

**DOI:** 10.1186/1471-2105-10-S1-S37

**Published:** 2009-01-30

**Authors:** Fei Xia, Yong Dou, Xingming Zhou, Xuejun Yang, Jiaqing Xu, Yang Zhang

**Affiliations:** 1 National Laboratory for Parallel&Distributed Processing, Department of Computer Science, National University of Defense Technology, ChangSha, 410073, PR China

## Abstract

**Background:**

In the field of RNA secondary structure prediction, the RNAalifold algorithm is one of the most popular methods using free energy minimization. However, general-purpose computers including parallel computers or multi-core computers exhibit parallel efficiency of no more than 50%. Field Programmable Gate-Array (FPGA) chips provide a new approach to accelerate RNAalifold by exploiting fine-grained custom design.

**Results:**

RNAalifold shows complicated data dependences, in which the dependence distance is variable, and the dependence direction is also across two dimensions. We propose a systolic array structure including one master Processing Element (PE) and multiple slave PEs for fine grain hardware implementation on FPGA. We exploit data reuse schemes to reduce the need to load energy matrices from external memory. We also propose several methods to reduce energy table parameter size by 80%.

**Conclusion:**

To our knowledge, our implementation with 16 PEs is the only FPGA accelerator implementing the complete RNAalifold algorithm. The experimental results show a factor of 12.2 speedup over the RNAalifold (*ViennaPackage *– 1.6.5) software for a group of aligned RNA sequences with 2981-residue running on a Personal Computer (PC) platform with Pentium 4 2.6 GHz CPU.

## Background

Ribonucleic Acid (RNA) is an important molecule that performs a wide range of functions in biological systems, such as synthesizing proteins, catalyzing reactions, splicing introns and regulating cellular activities. The function of an RNA molecule generally can be derived from its secondary structure. Currently, the only completely accurate method of determining the folded structure of an RNA molecule is by X-ray crystallography and nuclear magnetic resonance (NMR), however, those methods are time consuming and very expensive. Therefore, computational methods have been widely used in the field of RNA secondary structures prediction, such as thermodynamic energy minimization methods, homologous comparative sequences, stochastic context-free grammar methods (SCFG) and genetic algorithm and so on. Among which the most popular structure prediction algorithm is the Minimum Free Energy (MFE) method [1]. It was presented in 1981 by M. Zuker and has been implemented by three famous programs: Mfold [2], RNAfold [3] and RNAalifold [4] (the Vienna RNA package [5]).

Both Mfold and RNAfold implement the Zuker algorithm for computing minimal free energy (MFE) structures by folding a single sequence and employ the same thermodynamic parameters [6]. The time complexity is *O*(*n*^3^) and the spatial complexity is *O*(*n*^2^) by limiting the length of interior loops, where *n *is the sequence length. RNAalifold [4] implements an extension of the Zuker algorithm for computing a consensus structure from RNA alignments. The algorithm computes an averaged energy matrix and a covariation score matrix, augmented with penalties for inconsistent sequences. The algorithm requires extreme computational resources *O*(*m *× *n*^2 ^+ *n*^3^) in time, and *O*(*n*^2^) in space, where *n *is the sequence length and *m *is the number of sequences in the alignment [7].

Free energy minimization is the most common method for RNA secondary prediction. However, this method typically suffers two drawbacks. The first one is the limitation of structure prediction accuracy. The reason is that the thermodynamic rules are incomplete and the current model itself is an estimate of the real physics of RNA folding [8]. In practice, benchmarks of prediction accuracy on single RNA sequence show that current RNA folding programs get about 50–70% of base pairs correct on average [9]. The second one is the extreme demand for computational resources. The cost is intolerable with the growth in RNA database.

There are two kinds of parallel processing solutions based on MFE method at present, but both of them only considered the application of single sequence folding, Mfold and RNAfold, which are based on Zuke algorithm. High performance parallel computers with shared or distributed memory, such as SMP multiprocessor [10] or cluster systems [11] are widely used to accelerate Zuker algorithm [12,13]. The main idea is to partition the matrix in a regular fashion and to distribute tasks to multiple processors.

Unfortunately, the simple coarse-grain zone blocking method (1 million cells in a region) results in severe load imbalance because the size of the computation for each element is closely related with its position in the matrix. In [14-16], the authors presented some parallel implementations of the Zuker algorithm and methods for load balance. However, they did not consider communication delays, which account for 50% of the execution time for a sequence length 9212, due to fine grain data transfer. They achieve a 19× speedup on a 32-processor system, DAWNING 4000A [10], and 8× on a cluster with 16 Opteron processors running at 2.2 GHz, each with 3 GB memory [11]. The other solution to accelerate the Zuker algorithm is using multi-core architecture. Based on the IBM Cyclops64 simulator, G.M. Tan et al. [17] presented a parallel Zuker algorithm. They report a 30× speedup on 64 cores for an RNA sequence length of 2048. Parallel efficiency is greatly limited by complicated data dependency and tight synchronization. Thus, efficiently executing the MFE algorithm on a general-purpose computer or a multi-core architecture becomes very awkward.

Recently, the use of Field Programmable Gate-Array (FPGA) coprocessors has become a promising approach for accelerating bioinformatics applications. The computational capability of FPGAs is increasing rapidly. The top level FPGA chip from Xilinx Virtex5 series contains 51840 slices and 10368 K bits storage. The reconfigurability of FPGA chips also enables algorithms to be implemented with different computing structures on the same hardware platform.

Accelerating the MFE algorithm on FPGA chips is a challenging task. First, the non-uniform multi-dimensional data dependences with variable dependence distance make it difficult to find a well-behaved task assignment for load balance. Second, the irregular spatial locality with a great deal of small granularity access operations make it difficult to optimize memory scheduling for efficient external access. Third, multiple copies of free energy parameters for parallel processing consume a large amount of on-chip memory and memory ports limiting the physical scale of parallel processing. Finally, the limited on-chip memory cannot hold all *O*(*n*^2^) matrices, resulting in long-latency matrix loads from external Dynamic Random Access Memory (DRAM). So far, the algorithm accelerator based on MFE method is still under research. G.M. Tan et al. [18] introduced a fine-grained parallelization of the Zuker algorithm which considers only the interior loop calculation rather than the whole algorithm. A recent paper, Arpith Jacob et al. [19], implemented the simplest RNA folding algorithm, Nussinov algorithm [20], on a Virtex-II 6000 FPGA, but only input sequences with 30 ~ 60 bases can be predicted. Both presented results from simulation only.

In this paper, we propose a systolic array structure including one master PE and multiple slave PEs for fine grain hardware RNAalifold algorithm implementation on FPGA. We optimize the nested loop structure and reorganize the computation order by analyzing the data dependency in the original RNAalifold algorithm and improve the spatial locality in folding process. For load balance, we partition tasks by columns and assign tasks to PEs. We aggressively exploit data reuse schemes to minimize the need for loading energy matrices from external memory. Specifically, we add a cache to buffer a triangular sliding window of one of the matrices, most of which will be used in computing the next element in the column. We also transfer local elements directly to the next adjoining PE. In our design, only the master PE loads energy matrices from external DRAM. The remaining slave PEs simply wait for data from the previous PE. We also propose several methods that collectively reduce the storage requirements of the energy parameter tables by 80%-fitting curves with piecewise linear function, replacing scattered points with register constants and compressing the address space while shortening data length. The whole array structure is carefully pipelined in order to overlap multiple PE's column computations, master PE's load operations and multiple PE's write-back operations as much as possible. We implemented an RNAalifold algorithm accelerator with 16 processing elements on a single FPGA chip. The experimental results show a factor of 12× speedup over the *ViennaRNA*-1.6.5 software for a group of aligned RNA sequences with 2981-residue each running on a PC platform with Pentium 4 2.6 GHz CPU. Moreover, the power consumption is only about 1/8 of general-purpose microprocessor.

## Overview of the RNAalifold algorithm

### Brief introduction

The RNAalifold algorithm predicts a consensus secondary structure from a group of aligned RNA sequences by calculating an averaged minimum free energy for the alignment, incorporating covariance information into the energy model [21]. The essential idea of RNAalifold algorithm is still the thermodynamic energy minimization theory, which was first presented by M. Zuker in 1981. It uses a "nearest neighbor" model and empirical estimates of thermodynamic parameters for neighboring interactions and loop entropies to score structures [7]. The data input of RNAalifold is the result of multiple sequence alignment and the common secondary structure (base pairing result) will be generated through below three processing phases.

#### Calculating co-variance bonus

The function of this stage is calculating the co-variance bonus for each pair depending on compensatory or consistent mutations. Then it uses the bonus to judge if the residues located on the two positions can consist a base-pair and direct the energy filling process.

#### Filling energy matrices

**Figure 1:**
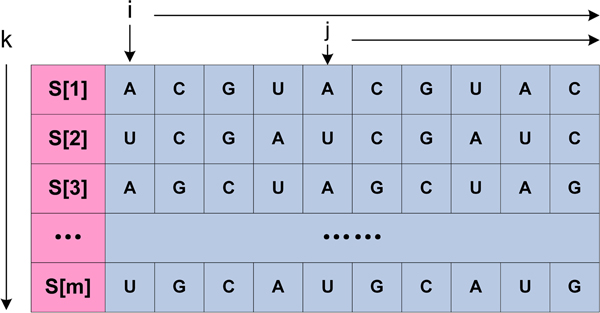
**An example of input sequences**. *S*[0], *S*[1], ..., *S*[*m*] represent a group of aligned RNA sequences. Where *i *and *j *represent the nucleotides' location in RNA sequence, *k *is the ID of current input sequence and *n *is the sequence length.

Suppose *S*[0], *S*[1], ..., *S*[*m*] is a group of aligned RNA sequences as shown in Figure 1. *r*_1_*r*_2_...*r*_*i*_...*r*_*j*_...*r*_*n *_is an RNA sequence, which consists of four nucleotides: A, C, G, U and a blank inserted by multiple sequence alignment tool, where *i *and *j *represent the nucleotides' location in RNA sequence, *k *is the ID of current input sequence and *n *is the sequence length.

**Figure 2:**
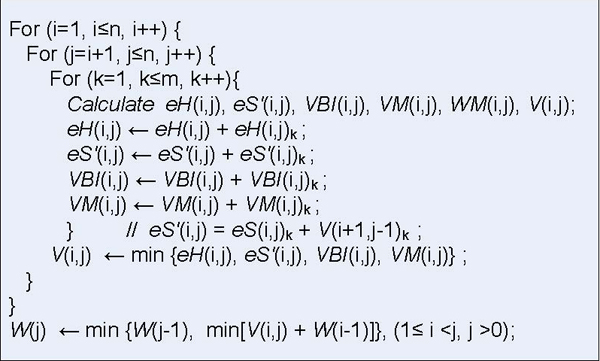
**The RNAalifold algorithm description**. This figure describes the core of energy matrices filling stage in RNAalifold.

The core of energy matrices filling stage in RNAalifold is a triple cycle operation as shown in Figure 2. The two control variables, *i *and *j *in surrounding loops, moves alone the horizontal axis to pass through every place and search for the potential base-pairs. The inner loop, control variable *k*, moves down and implements the free energy accumulation of substructure located on (*i*, *j*) position in different energy matrices. The value of *V*(*i*, *j*) equals the minimum value among the four energy parameters that four fundamental substructures corresponding respectively, which stands for the energy of an consensus optimal structure of the common subsequence *r*_*i*_...*r*_*j*_.

The vector *W *holds the minimal free energy for certain structures of common subsequences. The element, *W*(*j*), is the energy of a consensus optimal structure of the common subsequence *r*_1_*r*_2_...*r*_*j*_. Once the longest fragment, the complete sequence, is considered, the lowest conformational free energy is calculated then the filling step ends and *W*(*n*) stands for the energy of the most energetically stable structure of the aligned RNA sequences. The calculation of *W*(*j*) depends on its left elements from *W*(1) to *W*(*j*-1) and the *j*th column in matrix *V*, *V *(*, *j*).

**Figure 3:**
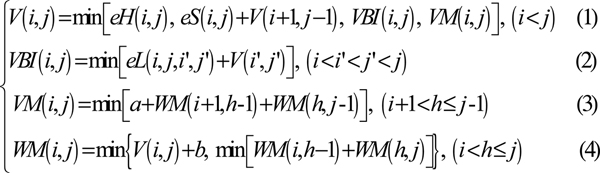
**The recurrence relations in RNAalifold**. The energy computing recurrence relations for each base-pair (*r*_*i*_·*r*_*j*_) in RNA sequence.

As for one of the input RNA sequence, *S*[*k*], the energy computing for each base-pair (*r*_*i*_·*r*_*j*_) involves four triangular matrices: *V*, *VBI*, *VM*, *WM *and three energy functions: *eS*(*i*, *j*), *eH*(*i*, *j*), *eL*(*i*, *j*, *i'*, *j'*). The recurrence relations as shown in Figure 3.

*V *(*i*, *j*) is the energy of the optimal structure of the subsequence *r*_*i*_*r*_*i*+1_⋯*r*_*j *_where *r*_*i*_*r*_*j *_comprises a base pair. *VBI*(*i*, *j*) is the energy of the subsequence from *r*_*i *_through *r*_*j *_where *r*_*i*_*r*_*j *_closes a bulge or an internal loop. *VM*(*i*, *j*) is the energy of the subsequence from *r*_*i *_through *r*_*j *_where *r*_*i*_*r*_*j *_closes a multi-branched loop. *WM *(*i*, *j*) is the energy of the subsequence from *r*_*i *_through *r*_*j *_that constitutes part of a multi-branched loop structure. *eS*(*i*, *j*), *eH*(*i*, *j*) and *eL*(*i*, *j*, *i'*, *j'*) are free energy functions, which are used to compute the energy of stacked pair, hairpin loop and internal loop respectively. In software folding solution, these free energy functions are calculated by looking up tables of the standard free energy parameters, which are detected by experimental method. The storage requirement of those tables is about 1 M Byte.

#### Backtracking

When the corresponding energy matrices of all input sequences have been filled out, the free energy for optimal consensus structure is known, which is stored in the element *W*(*n*), but the structure is unknown. The phase of backtracking is performed to determine the structure leading to the lowest free energy, using the free energies calculated in the filling step to revivify the exact structure. Experiments show that the energy matrices filling step consumes more than 99% of the total execution time. Thus, computing energy matrices quickly is critical.

### Characteristics of RNAalifold

We make five observations about the characteristics of the RNAalifold algorithm. These observations suggest details of the parallel implementation.

#### Observation 1. The computation size of each element in an energy matrix is variable and closely related with its position

Considering the most time-consuming calculation of matrix *V *for each input sequence, which is an upper triangle matrix as described in formula (1). The computation size (the number of add operations) for each element, *C*(*i*, *j*), is closely related with the indices *i *and *j*, as shown in formula (5).

(5) $$C(i,j)=(j-i+1)+\{\begin{array}{l} \frac{30\times 31}{2},j-i>30;\\ \frac{\left(j-i)\times (j-i+1\right)}{2},j-i\leq 30 \end{array}$$

The computation size of *jth *column *V*(*, *j*), *C*(*, *j*), is the sum of *C*(*i*, *j*) in the *jth *column:

(6) $$C(*,j)=\frac{j\cdot (j-1)}{2}+\{\begin{array}{l} \begin{array}{cc} S, & 3\leq j\leq 30; \end{array}\\ S+\frac{30\times 31\times (j-30)}{2},j>30; \end{array}$$

Where, *S *= 1 + 3 + 6 + ⋯ + $\frac{1}{2}$ × (*j *- 1) × (*j *- 2). The difference in computation size between column *j *and column *j *+ 1 is Δ*C*(*j*, *j *+ 1):

(7) $$\Delta C(j,j+1)=j+\{\begin{array}{l} \frac{j\times (j-1)}{2},3\leq j\leq 30;\\ \frac{30\times 31}{2},j>30; \end{array}$$

We can find that the computation size gradually increases with the matrix location moving up from bottom to top in the same column and increases with the location moving right in the same row. Specifically, the workload of *V*(1, *n*) is the heaviest one, it depends on the entire row and column elements of *WM *matrix and the bottom-left triangle region of *V*(1, *n*) with the maximum size $\frac{1}{2}$ × 30 × 31. This workload imbalance suggests a cyclic column allocation scheme, in which each processing element (PE) is assigned one column of matrix *V*. Each PE processes its column from bottom to top.

#### Observation 2. Parallel computation of *V *requires multiple copies of the free energy parameters

The calculation of each element of matrix *V *involves looking up parameter tables to get the values of the free energy functions *eH*, *eS *and *eL*, obtained from experimental methods. The tables are addressed by pairs of RNA residues. To calculate *V*(*i*, *j*), we first find the residue pair indexed by *i *and *j *in the RNA sequence, then lookup the tables to obtain the energy values. The number of query operations in RNAalifold for computing matrix *V *is *O*(*n*^3^), *n *is the RNA sequence size. For parallel computing, centralized tables will become the performance bottleneck. We have to distribute the parameter table to each PE so that energy values can be read without memory conflict. But the storage requirement of entire free energy parameter tables at 37°*C *is more than 128 KByte. In addition to other storage requirements for data buffers, the total storage requirement will greatly exceed the capacity of the current largest FPGA chip if the number of PEs is over 4. As a result, for RNAalifold, the storage factor has a major effect on the scalability of parallel processing.

We figured out several efficient compression approaches to reduce the storage overhead of the free energy parameters. First, we partition the loop destabilizing energies into segmented linear functions. The transformation from query operations to arithmetic operations reduces the requirements for memory ports and storage capacity. The linear functions are simple, require little logic, and have no impact on accuracy. Second, we represent a few scattered points using registers. Some parameters in free energy tables are scattered too widely to be fitted by simple linear functions. Instead of using block RAM to hold these parameters, we assign these parameters to registers. Third, we compress the address space and shorten the data length. Interior loop energy data occupies more than 80% of the total parameter tables, when four nucleotides, *A*, *C*, *G*, *U *are encoded directly. Instead, we encode the six base pairs, *AU*, *CG*, *CU*, *GC*, *UA *and *UC *using 3 bits. The table address length reduces from 16-bit occupying 64 K entries to 14-bit for 16 K entries. The storage requirement is compressed by 75%. Finally, we transform the raw data of free energy parameter tables from signed decimal fraction into complementary integer reducing data width from 16-bit to 8-bit without affecting accuracy. With the above schemes, the storage requirement of free energy parameter tables drops by 80%. As a result, more processing elements can be fitted in FPGA chip.

#### Observation 3. We can use a sliding window to reuse data within a column

**Figure 4:**
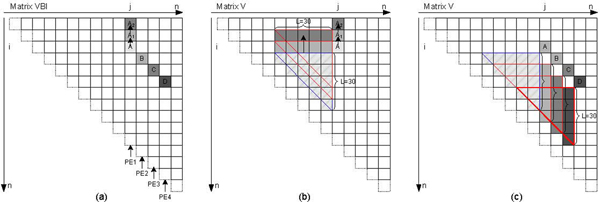
**The data dependence in RNAalifold**. (a) shows the elements of matrix VBI depend on a triangle window of matrix *V*; (b) shows the sliding window moving upward; (c) shows the data reusing between adjoining columns.

From formula (2), we observe the element *VBI*(*i*, *j*), which is used to calculate *V *(*i*, *j*), depends on the elements located in the bottom-left triangle region of *V *(*i*, *j*). As shown in Figure 4, the region is a triangle window with the maximum size of 30 × 30 due to the limitation of the interior loop length. In Figure 4, the blue triangle window in matrix *V*, called window A, contains the necessary data for computing the element A of *VBI*. With the computation traveling upward in the same column from *A*, *A*1, to *A*2, the triangle window moves from bottom to top.

We observe that only one row of elements is updated when the window slides from *A *to *A*1, the other elements remain unchanged. We use a local buffer of 465 elements ($\frac{1}{2}$ × 30 × 31) and prefetch 30 elements of *V *into it before calculating each element of *VBI*, saving 435 elements. This greatly reduces the memory bandwidth requirements for loading elements of *V *from external DRAM.

#### Observation 4. We can use a sliding window to reuse data between adjoining columns

The triangle windows in adjoining columns exhibit an overlapped area which can be exploited for additional data reuse. Assuming the four elements A, B, C and D located in four adjoining columns in Figure 4(c), we arrange four PEs, PE1, PE2, PE3 and PE4 for parallel computing, respectively. In Figure 4(c), we observe that triangle B contains two parts, one is a sub-triangle residing in triangle A completely, and the other is a column of A with a maximum size of 30 elements which becomes available before element A is computed. The same overlapped area can be found between B and C, C and D. This observation implies that except that the first column PE1 has to hold the entire triangle window, the other column PEs have to wait only for the elements transferred from the previous PE. A similar scenario is found in the computation of *WM*. As a result, by transferring data between the adjoining columns processing, we can greatly reduce the memory bandwidth requirements for loading elements of *V *and *WM *from external DRAM.

#### Observation 5. We can reorganize the computation order in parameter accumulating among multiple energy matrices to improve the spatial locality

**Figure 5:**
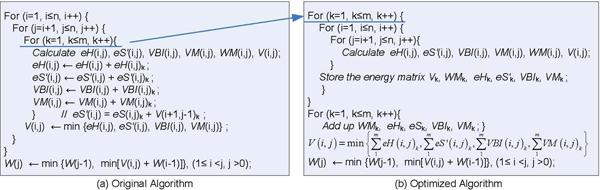
**The contrast between original and optimized RNAalifold algorithm**. (a) shows the kernel loops of energy filling stage in the original RNAalifold algorithm; (b) shows those of energy filling stage in the optimized RNAalifold algorithm. The main difference lies in the inner loop (variable *k*) of RNAalifold, which is brought out and the free energy computation (the *Calculate *statement, which implements the recurrence relations (1)~(4)) and accumulation (the 4 assignment statements) process are separated. In optimized algorithm, the free energy matrices of each RNA sequence corresponding are calculated and stored by executing the triple For-loop operations at first. Then, the independent For-loop statement implements the accumulation operation for adding up the free energy parameters for all input sequences. At last, the matrices *V *and *W *are calculated for backtracking.

In order to recover the consensus structure of the common subsequences from position *i *to *j *in a group of aligned RNA sequences, the sum of energy value of all fragments located in the same position (*i*, *j*) must be figured out. In the original RNAalifold algorithm, the sum-of-energy is calculated by adding up the energy of the same position in all input sequences one by one (the parameter *k *increases from 1 to *m *in the inner loop as shown in Figure 5(a)). According the recurrence relations (1)~(4) and the observations 3 and 4, the parameter *V*(*i*, *j*) of each sequence corresponding is depend on its energy matrices *V *and *WM*.

The jumping of current energy computing and accumulation operand among the multiple energy matrices will cause the high-frequency data exchanging (RNA sequences and the elements located in sliding triangle window in every energy matrix *V*) between FPGA and off-chip memory. As a result the poor spatial locality and low efficiency of external memory access will become the performance bottleneck in FPGA implementation.

To address the problem, we improved the nest relationship of the triple cycle operation in original algorithm. In the original algorithm as shown in Figure 5(a), only the current computing result, the four energy components on position (*i*, *j*) (*eH *(*i*, *j*)_*k*_, *eS' *(*i*, *j*)_*k*_, *VBI *(*i*, *j*)_*k*_, *VM *(*i*, *j*)_*k*_), can be reused.

However, in the optimized algorithm, the elements located in the triangle region of *V *(*i*, *j*) and current rowcolumn of *WM *(*i*, *j*) can be reused for computing the next elements *V *(*i *- 1, *j*) and V (*i*, *j *+ 1). As a result, we can eliminate the high-frequency data exchanging between FPGA and off-chip memory and improve the access efficiency of external memory.

## Methods

### System architecture

**Figure 6:**
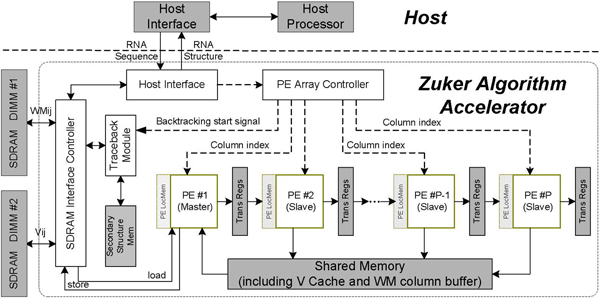
**The structure of RNAalifold algorithm accelerator**. This figure describes the structure of our RNAalifold algorithm accelerator. The accelerator comprises one FPGA chip, two SDRAM modules, and one I/O channel to the host PC.

Our RNA folding computation platform consists of an algorithm accelerator and a host processor. The accelerator receives a group of aligned RNA sequences with a co-variance bonus matrix, executes the energy matrices filling and backtracking phases, and reports the consensus RNA secondary structure represented in base pairs back to the host for display. The structure is shown in Figure 6.

The accelerator engine comprises one FPGA chip, two SDRAM modules, and one I/O channel to the host PC. Two SDRAM DIMMs store the energy matrices of each sequence for energy accumulating and backtracking, and are connected to the FPGA pad directly. The host interface channel is responsible for transferring initial RNA data, co-variance bonus and final results between the accelerator and the host. The core of the RNAalifold algorithm accelerator is composed of a PE Array Controller, a PE array, a Shared Memory Module, an Energy Matrices Superposition Module and a Trace-back Module. The PE Array Controller is responsible for assigning column tasks to the PE array and switching from the filling phase to the backtracking phase. The Shared Memory module contains a *V *cache, which holds the triangular sliding window and a *WM *column buffer, which stores the current *p *column elements for writing back to SDRAM, where *p *is the number of PEs. The PE array performs the free energy calculation in parallel. The array consists of a series of PE modules, in which the first one, PE1 is the master and the others are slaves. Each PE is augmented with a local memory to store a copy of the current RNA sequence and a register for the current column elements of matrix *WM*. The registers between adjoining PEs, called the Trans Regs, are used for delivering reusable data including *WM *row/column elements and the bottom-left elements of *V*(*i*, *j*). The Energy Matrices Superposition Module is responsible for energy accumulating and generating the energy matrices (*V*, *WM*, *W*) for backtracking.

### Master-slave PE array algorithm

**Figure 7:**
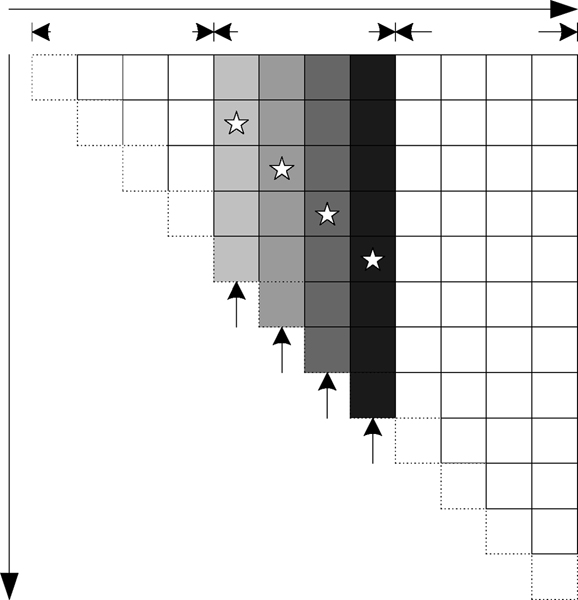
**The task partitioning and the computation order with 4-PE array**. There are 4 dark columns assigned to PE1 to PE4 in the middle area representing the current section. The positions belonging to the same diagonal marked with stars represent the current computation points of the 4 PEs. The parallel RNAalifold algorithm is divided into three phases, energy calculation, columns synchronization and section advance.

**Figure 8:**
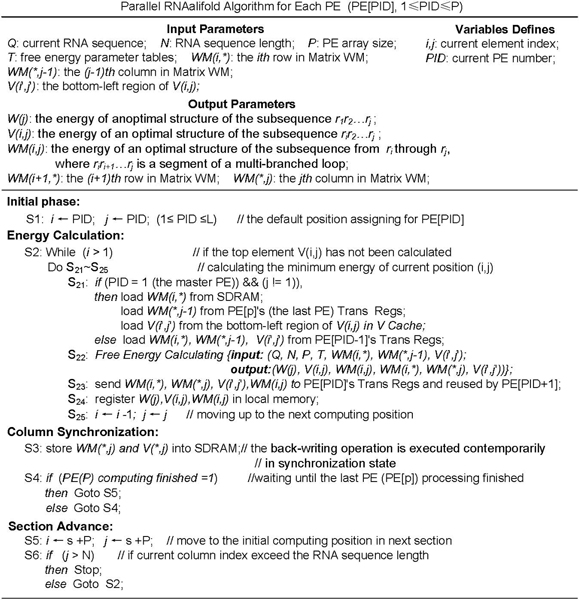
**The master-slave PE array algorithm**. The first part of Figure 8 shows the parameter and variable definitions. At the initial phase, each PE assigns its PE identifier, PID, to the column index indicating the initial column assignment. Before the energy calculation phase several data items are loaded into local memory as shown in S21. Only the master PE loads data from SDRAM and the *V *cache, the other slave PEs only load data from Trans Regs of the previous adjoining PE. When the energy data are ready in local memory, the free energy calculation is performed as shown in S22. From S23 to S25, all PEs transfer data received from the previous adjoining PEs to their own Trans Regs, store results to local memory, and move the computation point upward in the same column. When the computation point moves to the top of the current column, the PE enters the column synchronization phase, during which it writes its local results to SDRAM. When all PEs arrive at the synchronization points, indicating the end of the current section, the section advances by each PE adding *p *to its column index. The process repeats until the column index is greater than the RNA sequence length.

Free energy computing (the Calculate statement in the triple For-loop as shown in Figure 5) is the kernel in the RNAalifold algorithm. As implied by observation 1, the upper-triangular shaped energy matrices are partitioned into columns. Each PE holds one column cyclically in turn. Every group of *p *contiguous columns forms a section as shown in Figure 7. Figure 8 describes the parallel RNAalifold algorithm in a Single-Program Multiple-Data (SPMD) style.

### Performance measures

The execution time of our parallel RNAalifold algorithm can be predicted in cycles due to the tight synchronization of the systolic array structure. The total execution time (*T*) is the sum of matrices filling time (*T*_*f*_), matrices accumulating time (*T*_*a*_) and the time for trace-back (*T*_*t*_). Moreover, the matrices filling time (*T*_*f*_) equals the energy computation time (*T*_*c*_) plus the external memory access time (*T*_*m*_). Assuming *p *is the number of PEs, *n *is the length of the aligned RNA sequence, *m *is the number of input sequences and *s *= $\left\lfloor \frac{N}{p}\right\rfloor$, we have energy computation time (*T*_*c*_) in (8)

(8) $$T_{c}=\frac{1}{4}p\cdot \Delta t_{1}\cdot \left[\frac{s(s+1)}{2}+\frac{s(s+1)(2s+1)}{6}p\right]$$

The time for accessing external memory (*T*_*m*_) is *T*_*m *_= $\frac{n\cdot (n+1)}{2}$·Δ*t*_2_. Thus, the filling time for all energy matrices (*T*_*f*_) is

(9) $$T_{f}=\frac{m\cdot p\cdot \Delta t_{1}}{4}\cdot \left[\frac{s(s+1)}{2}+\frac{sp(s+1)(2s+1)}{6}\right]+\frac{1}{2}\cdot m\cdot n\cdot (n+1)\cdot \Delta t_{2}$$

The matrices accumulation time (*T*_*a*_) is $\frac{(m-1)\cdot n^{2}}{2}$·Δ*t*_1 _and the time for trace-back (*T*_*t*_) is *T*_*t *_= $\frac{3n^{2}}{8}$. Where Δ*t*_1 _is the time for each sum operation, which is one cycle in our implementation, Δ*t*_2 _is the average access overhead for storing an element to external memory, which is 15 cycles in the worst case. As a result, the total execution time (*T*) is

(10) $$T=\frac{mp}{4}\cdot \left[\frac{s(s+1)}{2}+\frac{s(s+1)(2s+1)}{6}\cdot p\right]+\frac{15mn\cdot (n+1)}{2}+\frac{(m-1)\cdot n^{2}}{2}+\frac{3\cdot n^{2}}{8}$$

According to the formula (9) and (10), we can theoretically analyze the parallel efficiency (*E*_*c *_= $\frac{1}{1+\alpha }$) of our accelerator, where *α *is

(11) $$\alpha =\frac{3n^{2}(4m-1)}{smp(s+1)(2sp+p+3)+180mn(n+1)}$$

In the general case that *m *= 100, *p *< 128 and *n *> 1024, the parameter *α *is always less than 0.1. As a result, the parallel efficiency can reach more than 90%, showing good parallelism.

## Results and discussion

### Experiment environment

We implement the RNAalifold algorithm accelerator in an FPGA testbed. The testbed is composed of one FPGA chip, StratixII EP2S130C5 from Altera, two 1 GB SDRAM modules, MT16LSDT12864A from Micron and a USB2.0 interface to the host computer. The folding software, RNAalifold (*ViennaPackage*-1.6.5 download from Vienna RNA web site [22]), runs on a desktop computer with Intel Pentium4 2.60 GHz CPU and 1 GB Memory at level *O*3 compiler optimization. Both the accelerator and software use the same free energy parameters, RNA free energies at 37°*C*, Version 3.0 downloaded from M. Zuker's homepage. We also measure software execution time on AMD and Xeon platforms to verify the acceleration of our approach.

### Verifying correctness

We verify the correctness of our implementation in three steps. First, we ensure the correctness of the optimized algorithm by comparing the software folding result. Second, we implement hardware RNAalifold algorithm and verify the function correctness of the hardware using software emulation with ModelSim SE 6.2 h. Then, we run the search in our testbed to compare the base-paring results with the ones produced by software to verify the correctness of the folding result generated by our accelerator. We fold six group of RNA sequences (20 sequences in each group) with the size from 83 bps to 2981 bps by using hardware and software solutions respectively. The experimental results show that the folding results of our accelerator agree with the software version.

### Resource usage

Besides implementing the accelerator on Altera FPGA chips, we place different numbers of PEs on Xilinx FPGA chips to evaluate the resource usages as well. As shown in the last row of Table 1, in Altera FPGA chips, one PE consumes 3817 ALUTs and 332 K bits of memory. It consumes 2124 slices and 2013 slices in Xilinx XC4V and XC5V FPGA chips respectively. At most 16 PEs fit on EP2S130C5 because the storage requirement consumes almost all of the memory resources. On XC5VLX330, the latest FPGA from Xilinx, we can fit 20 PEs. All implementation can reach a clock frequency of over 130 MHz.

**Table 1:** Resource usage on different FPGA platforms

FPGA	EP2S130C5	XC4VLX200-11	XC5VLX330-2
PE Fitted	16	16	20

ALUTSlice (%)	75720 (71%)	46483 (52%)	42097 (80%)

Total Memory (%)	6328 Kb (96%)	6592 Kb (88%)	11828 Kb (89%)

Clock (MHz)	133	135	138

Single PE	3817 ALUTs332Kb	2124 Slices	2013 Slice

### Scalability

**Figure 9:**
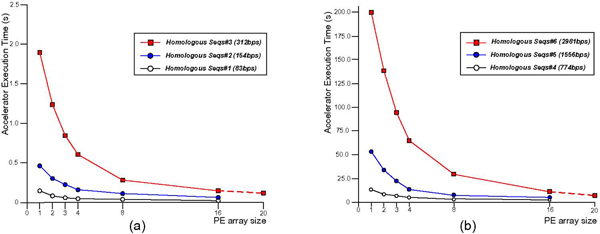
**The execution time (s) on different PE array size for different queries**. The horizontal axis represents PE array size and the vertical axis represents execution time of hardware accelerator. The six curves with different color represent input RNA sequences with different length.

**Figure 10:**
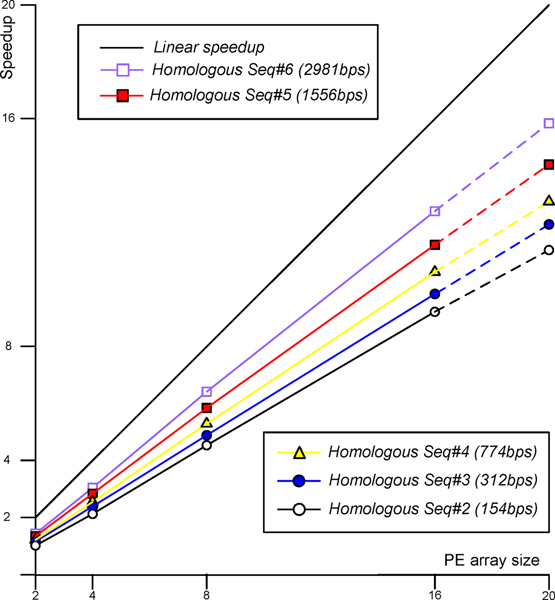
**The speedup on different PE array size for different queries**. The horizontal coordinate represents PE array size and the vertical coordinate represents the speedup of each group of input RNA sequences.

To explore the scalability of the proposed accelerator architecture, we folded six group of sequences on our accelerator. As shown in Figure 9, the execution time of input RNA sequences with varying length from 83-base to 2981-base on multiple PEs includes computation time, trace-back time and the time for sending sequence query and taking results back for display. Because execution time greatly increases with the increase in sequence length ranging from 0.15s for 83 bases to 199.8s for 2981 bases, we show the execution time in different figures. Considering the longest sequence with 2981 bases, the execution time is shortened sharply from 199.8s for one PE to 16.4s for 16 PEs, a factor of 12.2 speedup. Performance with 20 PEs is estimated according to the formula (10). Figure 10 shows the speedup with different sequence lengths ranging from 83 to 2981 exhibit similar linear features due to the scalable parallel structure in the accelerator.

### Performance compared to ViennaRNA

The original loops in RNAalifold are unrolled and the calculation order in energy matrices filling stage is reorganized in our fine-grained parallel algorithm. However, the time complexity is undiversified since the number of add and compare operations are not change. In the original algorithm, only two triangle matrices (*V *and *WM*) are stored for each sequence in energy matrices filling stage. In optimized algorithm, the other four substructure matrices (*eS*, *eH*, *V BI*, *VM*) are also stored for energy accumulation. Thus, the parallel algorithm needs triple storage requirement compared to the original RNAalifold software. In order to reduce the bandwidth requirements for external memory access, the six triangle matrices are compressed into two matrices by using component combined strategy. Furthermore, the data reusing in PE array is well exploited with the spatial locality improving. Therefore, the increasing of storage requirements for off-chip memory will not become the bottleneck in FPGA implementation. Taking Pentium 4 as the base, we compare the execution time and speedup among 4 different platforms, including three general-purpose computers and our algorithm accelerator. Despite the variation in CPU type, clock frequency, main memory capacity, cache capacity and software versions, the three general-purpose computers exhibit similar performance. As shown in Table 2, Athlon shows a little advantage over Pentium and Xeon, achieving at most 1.4× speedup. However, the FPGA accelerator exhibits significant speedup ranging from 8.4 to 12.2.

**Table 2:** Execution time(s) and speedup with different input on different platforms

Sequence Size (M = 20)	N = 154		N = 387		N = 1556		N = 2981	
	
	Time	Speedup	Time	Speedup	Time	Speedup	Time	Speedup
FPGA(16-PE)	0.05	8.4	0.19	8.7	5.1	9.8	16.4	12.2

Pentium^(1)^	0.42	1	1.65	1	49.5	1	199.8	1

Xeon^(2)^	0.38	1.1	1.38	1.2	45.1	1.1	178.5	1.1

Athlon^(3)^	0.31	1.4	1.18	1.4	37.8	1.3	141.6	1.4

The soft/hardware experimental environment of different platforms are listed as follows. Pentium^(1)^: 2.6 G CPU, 1.0 GB memory, 512 KB Cache, Linux 2.6.5, GCC 4.1.0; Xeon^(2)^: 2.8 G CPU, 1.0 GB memory, 1024 KB Cache, Linux 2.4.2, GCC 3.2.3; Athlon^(3)^: 2.0 G CPU, 2.0 GB memory, 512 KB Cache, Linux 2.6.18, GCC 4.1.1;

### Power consumption compared to general-purpose microprocessor

FPGA accelerator is also energy-efficient relative to general-purpose computers. Our RNAalifold algorithm accelerator with 16 PEs only consumes 9.2 *W *as simulated by Xilinx ISE 9.2 XPower tool. General-purpose microprocessors consume between 80 *W *to 150 *W *on average [23]. Furthermore, considering the low clock frequency of 130 MHz in FPGA chips, we believe the application-specific fine-grained schemes implemented in our accelerator provides significant advantage over the general-purpose schemes.

## Conclusion

The minimum free energy (MFE) method plays an important role in the area of RNA secondary structures prediction. Many parallel implementations on general purpose multiprocessors exhibit parallel efficiency of no more than 50%. In this paper, we explore the use of FPGAs to accelerate the RNAalifold algorithm based on MFE method.

After carefully studying the characteristics of the algorithm, we make five observations to direct our design. We optimize the nested loop structure in original RNAalifold and reorganize the computation order to improve the spatial locality. We propose task assignment in cyclic column order to achieve load balance. We introduce two data reuse schemes that use a sliding window cache and transfer registers between adjoining PEs. We also presented several methods to reduce the storage requirement for holding multiple copies of energy parameter tables. The experimental evaluation demonstrates that the performance of our algorithm accelerator is scalable with multiple PEs and that the FPGA accelerator outperforms general-purpose computers with a speedup of more than 12× on 16 PEs.

## Competing interests

The authors declare that they have no competing interests.

## Authors' contributions

Fei Xia carried out the fine-grained parallel RNAalifold algorithm, participated in the characteristics analysis of the RNAalifold algorithm and drafted the manuscript. Yong Dou conceived of the study, and participated in its design and helped to draft the manuscript. Xingming Zhou and Xuejun Yang participated in the discussion of the study and performed the performance evaluation. Jiaqing Xu participated in the sequence alignment and the analysis of original RNAalifold algorithm. Yang Zhang participated in hardware implementation and correctness verification and power consumption analysis. All authors read and approved the final manuscript.
